# Electron Beam Welding of IN792 DS: Effects of Pass Speed and PWHT on Microstructure and Hardness

**DOI:** 10.3390/ma10091033

**Published:** 2017-09-05

**Authors:** Giuliano Angella, Giuseppe Barbieri, Riccardo Donnini, Roberto Montanari, Maria Richetta, Alessandra Varone

**Affiliations:** 1 Institute of Condensed Matter Chemistry and Technologies for Energy (ICMATE), National Research Council of Italy (CNR), 20125 Milan, Italy; giuliano.angella@cnr.it (G.A.); riccardo.donnini@cnr.it (R.D.); 2 ENEA, Department for Sustainability-Research Centre of Casaccia, Santa Maria di Galeria, 00123 Rome, Italy; giuseppe.barbieri@enea.it; 3 Department of Industrial Engineering, University of Rome Tor Vergata, 00133 Rome, Italy; richetta@uniroma2.it (M.R.); alessandra.varone@uniroma2.it (A.V.)

**Keywords:** Ni base superalloy, IN792 DS, electron beam welding, post-welding heat treatments, microstructure

## Abstract

Electron Beam (EB) welding has been used to realize seams on 2 mm-thick plates of directionally solidified (DS) IN792 superalloy. The first part of this work evidenced the importance of pre-heating the workpiece to avoid the formation of long cracks in the seam. The comparison of different pre-heating temperatures (PHT) and pass speeds (*v*) allowed the identification of optimal process parameters, namely PHT = 300 °C and *v* = 2.5 m/min. The microstructural features of the melted zone (MZ); the heat affected zone (HAZ), and base material (BM) were investigated by optical microscopy (OM), scanning electron microscopy (SEM), energy dispersion spectroscopy (EDS), electron back-scattered diffraction (EBSD), X-ray diffraction (XRD), and micro-hardness tests. In the as-welded condition; the structure of directionally oriented grains was completely lost in MZ. The γ’ phase in MZ consisted of small (20–40 nm) round shaped particles and its total amount depended on both PHT and welding pass speed, whereas in HAZ, it was the same BM. Even if the amount of γ’ phase in MZ was lower than that of the as-received material, the nanometric size of the particles induced an increase in hardness. EDS examinations did not show relevant composition changes in the γ’ and γ phases. Post-welding heat treatments (PWHT) at 700 and 750 °C for two hours were performed on the best samples. After PWHTs, the amount of the ordered phase increased, and the effect was more pronounced at 750 °C, while the size of γ’ particles in MZ remained almost the same. The hardness profiles measured across the joints showed an upward shift, but peak-valley height was a little lower, indicating more homogeneous features in the different zones.

## 1. Introduction

Ni base superalloys are used in several industrial sectors such as spacecrafts, energy production (extraction of oil and gas, nuclear reactors, etc.), and piping for heat exchangers [1,2]. Of particular relevance is their use in components high temperature applications such as disks and the blades of aeronautic engines and gas turbines [3,4,5,6,7]. Their microstructure mainly consists of particles of the L12 ordered Ni_3_(Al,Ti) γ’ phase embedded in the disordered γ phase matrix [8]. The excellent stability of the (γ + γ’) microstructure depends on the misfit of the lattice parameter between γ’ and γ phases, and on the reduced interface energy (20–30 mJ/m^2^) which results from a strict control of the chemical composition and the parameters of production process.

Since mechanical parts making of these materials operate under high stress and temperature and in aggressive environments, damage may occur and accumulate with consequent degradation of mechanical properties. Fatigue and creep at high temperature induce precipitation of undesired phases, coalescence of γ’ precipitates, and morphological evolution of carbides; additional damage comes from oxidation and corrosion and consists of cracks which are generally repaired through joining techniques [9]. Welding is a hard task because there are strict requirements, namely (i) little modification of the original microstructure; (ii) no relevant residual stresses in the molten (MZ) and heat affected (HAZ) zones; (iii) no cracks in the MZ and HAZ; (iv) no massive surface segregation; (v) low elemental diffusion that changes the chemical composition of γ and γ’ phases.

The microstructure of the MZ in a joint is strongly affected by two phenomena occurring during solidification, i.e., dendritic growth, and solute partitioning with consequent formation of carbides, borides and other metallic compounds. Furthermore, low melting compounds may give rise to micro-cracks after post-welding heat treatments (PWHTs) [10], and local residual stresses arise in the MZ, leading to failure of mechanical parts [11,12].

A lot of work has been devoted in the past decades to the joining of Ni base superalloys and different techniques have been developed: Transient Liquid Phase Bonding (TLP) [13], Activated Diffusion Bonding (ADB) [14], and Brazing Diffusion Remetalling (BDR) [15]. Recently, attention has been focused on the high energy density techniques, like laser [16] and electron beam (EB) [17] because, in comparison with other techniques, they produce narrower seams with enhanced penetration depth, and involve lower heat input with the consequent reduction of MZ, HAZ, and residual stresses [18,19,20,21]. Compared to other welding techniques, EB permits maximum energy concentration (>10^7^ W/cm^2^), and the operations are carried out in a high vacuum, therefore a further advantage is the low gas absorption by liquid metal, and the intrinsic protection of the melt pool from atmospheric gases. Laser has a comparable energy concentration (>10^6^ W/cm^2^), but requires great attention to protect the melt pool.

In the last years, the great interest for 3D metal printing highlighted the characteristics of selective laser melting (SLM), a technology that allows the processing of different alloys including Ni base superalloys. The product is built by melting selected areas of powder layers under a protective atmosphere: under computer control, a high intensity laser beam selectively scans a powder bed, melting the particles which solidify to form a thin layer. Reference works can be found in [22,23,24].

The present work reports the results obtained on the IN792 directionally solidified (DS) alloy welded by EB in different operative conditions and submitted to PWHT. This alloy is widely used in aircraft turbines because of its good hot corrosion resistance and excellent strength at high temperature; however it often suffers hot cracking, i.e., crack formation in the final stage of solidification [25,26].

This results from the competition between shrinkage stress during cooling and intrinsic material strength, and it has been found that liquation is the primary cause of low HAZ crack resistance in most Ni-base superalloys. The precipitation associated with microfissures and grain boundaries in HAZ around EB welds in a similar alloy, IN 718, was described by Vincent [27]. Microstructure in HAZ of the same alloy were investigated by Huang et al. [28], while Ferro et al. [29] studied the influence of weld heat input on the joint microstructure in Inconel 706.

EB has been used to produce seams on 2 mm thick plates of IN792DS, with and without pre-heating of the workpieces, and by changing the pass speed. To remove residual stresses, the joints were then submitted to PWHT at 700 and 750 °C with a holding time of 2 h. The microstructural features of MZ, HAZ and BM have been investigated by optical microscopy (OM), scanning electron microscopy (SEM), energy dispersion spectroscopy (EDS), electron back-scattering diffraction (EBSD), X-ray diffraction (XRD) and micro-hardness tests.

## 2. Material and Methods

The chemical composition of IN792 DS is reported in Table 1.

The as-cast alloy was solubilized at 1120 °C for 2 h in a vacuum, aged at 845 °C for 12 h, and cooled in air to room temperature. The material was supplied in form of cylindrical ingots (Φ = 25 mm, L = 60 mm) with the [100] crystallographic direction of the oriented grains parallel to the ingot axis. As described in detail in [30], plates with thickness of 2 mm were obtained by spark-erosion cutting along the ingot axis. The solidification structure along the ingot axis was examined by macro-etching with Marble’s reagent (10 g CuSO_4_ + 50 mL H_2_O + 50 mL HCl).

**Figure 1:**
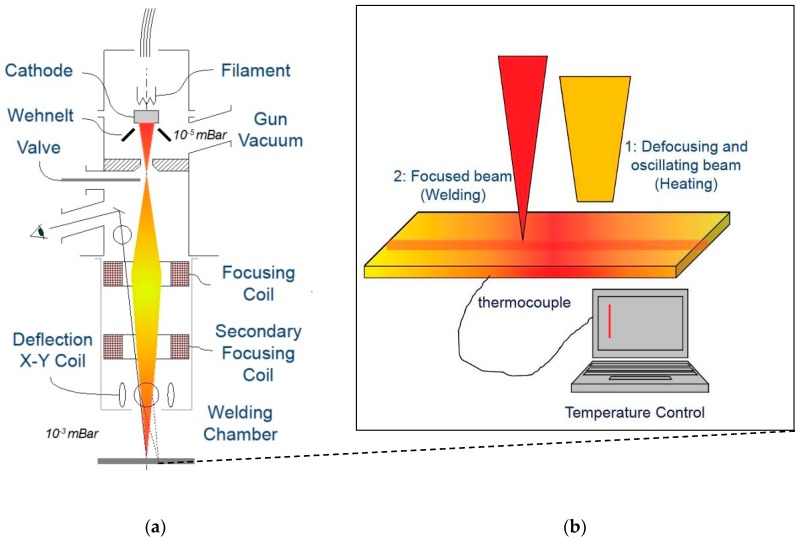
Sketch of the apparatus used for realizing electron beam (EB) welds (**a**). Detail of the workpiece (**b**): a thermocouple measures the temperature of the material that is pre-heated by defocusing and wobbling the electron beam.

After mechanical polishing and degreasing, welding operations were performed by using a Techmeta gun (Techmeta, Tessy, France), model CT4 with maximum power of 50 kW and maximum voltage of 80 kV. The welding chamber was 1.3 m^3^ (length 1300 mm; width 1000 mm; height 1000 mm) and contained the system for positioning and translating the parts to be welded. The experiments were performed both at room temperature and with pre-heating of the plates at 200 and 300 °C. Pre-heating was made by defocusing and wobbling the electron beam. A sketch of the apparatus is reported in Figure 1.

Preliminary tests were carried out to identify optimal focussing and power to reach full penetration in the condition of 1 m/min, threshold speed for high energy density processes. The successive tests were aimed to optimize pass speed, pre-heating temperature (PHT), and PWHTs. The seams on the sample plates were carried out longitudinally (parallel to the [100] direction), varying the pass speeds (1.0, 1.5, 2.0 and 2.5 m/min) and the other process parameters kept constant: power = 1 kW, acceleration voltage = 50 kV, beam current = 20 mA. The welded samples were then submitted to PWHT at 700 and 750 °C for 2 h.

After a preliminary check by X-ray radiography (GE Sensing & Inspection Technologies, Ahrensburg, Germany) to identify possible macro-defects, such as porosities, cracks etc., the joints before and after PWHT were examined by OM and SEM. In particular, to investigate morphology, size and distribution of γ and γ’ phases it was used a high-resolution SEM Hitachi SU70 (Hitachi, Tokyo, Japan) with TFEG source equipped with EDS and EBSD system Noran 7 by ThermoFisher Scientific (Waltam, MA, USA).

Disks (diameter of 3 mm, thickness of 100 µm) were prepared by mechanical polishing, then electrolytically etched using a twin jet system with a solution of 10% perchloric acid in methanol (−30 °C and 5 V). The sample surfaces of MZ and HAZ for EBSD observations were prepared through conventional metallographic preparation with final colloidal silica polishing, and EBSD patterns were gathered by using an acceleration voltage of 30 kV to minimize the disturbance effects from the surface defects introduced by sample preparation.

**Figure 2:**
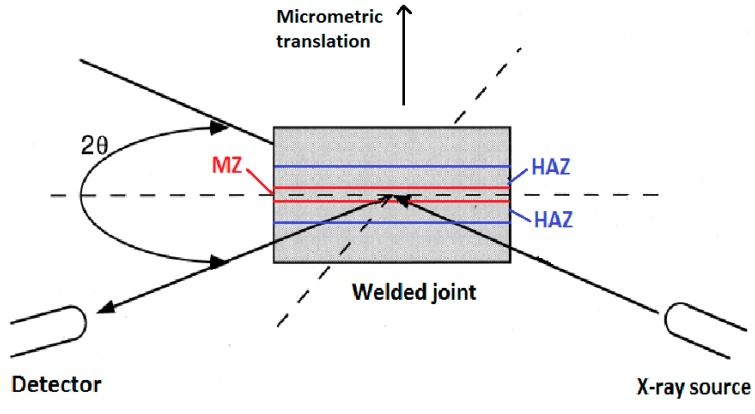
Experimental set-up used for focusing the X-ray beam on base metal (BM), heat affected zone (HAZ) and molten zone (MZ) of the joints.

XRD (Philips, Eindhoven, The Netherlands) measurements were carried out by focusing the X-ray beam on base metal (BM), HAZ, and MZ in order to determine the corresponding fraction of γ’ phase. The samples were mounted onto a micrometric translating table that allowed to move the samples and irradiate the desired zone. A sketch of the experimental set-up is shown in Figure 2. The {110} superlattice and {220} fundamental reflections were collected by using the Mo-Kα radiation (γ = 0.07093 nm), with 2θ angular intervals of 0.005° and counting times of 50 s per step. Therefore, the total amount ζ of γ’ phase was calculated from the following relationship [31]:

(1) $$\zeta =\frac{F_{220}^{2}L_{220}}{F_{110}^{2}L_{110}}\left(\frac{I_{110}}{I_{220}}\right)$$

where *F* is the structure factor, *I*-the integrated intensity and *L*-the Lorentz-polarization factor of {110} superlattice and {220} fundamental reflections. The *L*-factor depends on the Bragg angle θ and is given by:

(2) $$L=\frac{1+\cos^{2}2\theta }{\sin^{2}\theta \cos\theta }$$

In this operational condition the amount of γ’ phase was determined with an experimental error of about ±5%.

For hardness profiles across the weld seams, Vickers micro-hardness tests were conducted by using a load of 500 g.

## 3. Results and Discussion

### 3.1. The Base Metal

**Figure 3:**
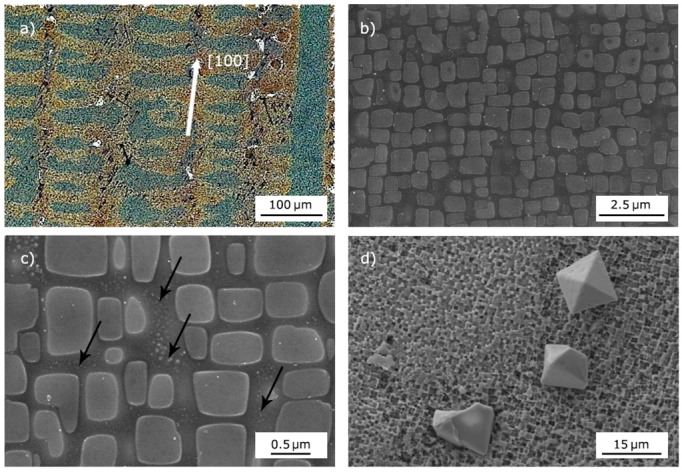
Directional structure of the alloy with [100] crystallographic direction parallel to the ingot axis (**a**); scanning electron microscope (SEM) micrographs (secondary electron imaging) of γ’ cube particles in the matrix (**b**); secondary γ’ particles (indicated by black arrows) of round shape and small size are observed in the channels between γ’ cubes (**c**); faceted Ti-Ta carbides (**d**).

**Figure 4:**
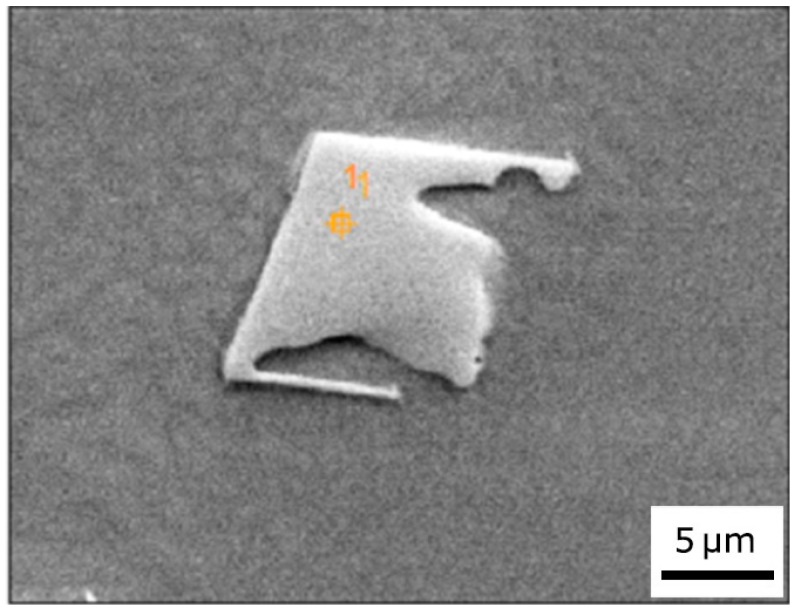
Energy dispersion spectroscopy (EDS) data (average of 10 measurements) reveal that it is rich in Ti and Ta.

In Figure 3a, macro-etching of the as-received material showed columnar grains typically extended along the full length of the ingot and consisting of numerous dendrites parallel to the solidification direction. Average primary and secondary dendrite arms spacing was approximately 120 and 70 μm, respectively. From image analysis, the volume fraction of the ordered γ’ phase was about 70%, the cubic particles had a mean size of ~400 nm, and were separated by channels of ~50–150 nm (Figure 3b). Embedded in the matrix between γ’ cubes, other particles of secondary γ’ phase with a round shape and smaller size (20–40 nm) were observed (Figure 3c). Figure 3d shows the presence of faceted particles with a mean size of about 10 μm. They were identified as Ti-Ta carbides from shape, elemental composition and literature data (e.g., see [32]). EDS data (average values of 10 measurements) collected from one of them are reported in Figure 4.

### 3.2. EB Welding Without Pre-Heating

**Figure 5:**
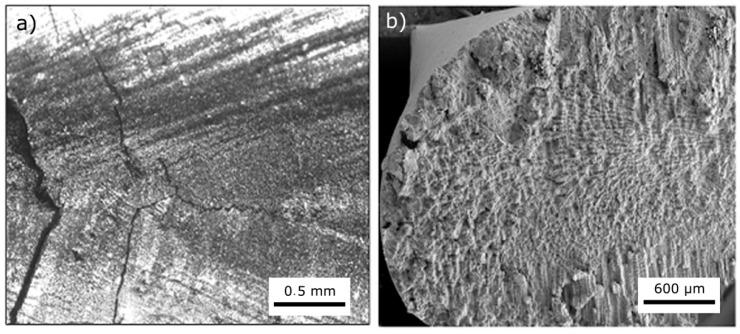
Sample welded without pre-heating: cracks and pores are observed in the seam (**a**); fracture surface clearly shows the dendritic structure (**b**).

A first group of experiments was performed by welding without pre-heating (PHT) to join the plates. EB seams always presented macro-defects like cracks and porosity (Figure 5a). The cracks start from the seam centre, initially move along its axis, then divert of about 45°. SEM observations of broken surfaces in the seam (Figure 5b) clearly showed that the cracks followed the interdendritic boundaries, a typical feature of hot tearing. High cooling rates favor hot tearing because they increase the temperature range of solidification leading to sub-solidus interdendritic liquid films and depress the solid state diffusion, fundamental for preventing microsegregation. The combination of shrinkage stresses and micro-segregation arising from the solidification of the liquid between secondary dendritic arms leads to crack formation and its successive propagation along the easier path, i.e., the interdendritic boundaries. This phenomenon is favored by high thermal gradients arising in EB welding, thus the following EB welding tests were performed by pre-heating the samples at PHT of 200 and 300 °C.

### 3.3. EB Welding with Pre-Heating

**Figure 6:**
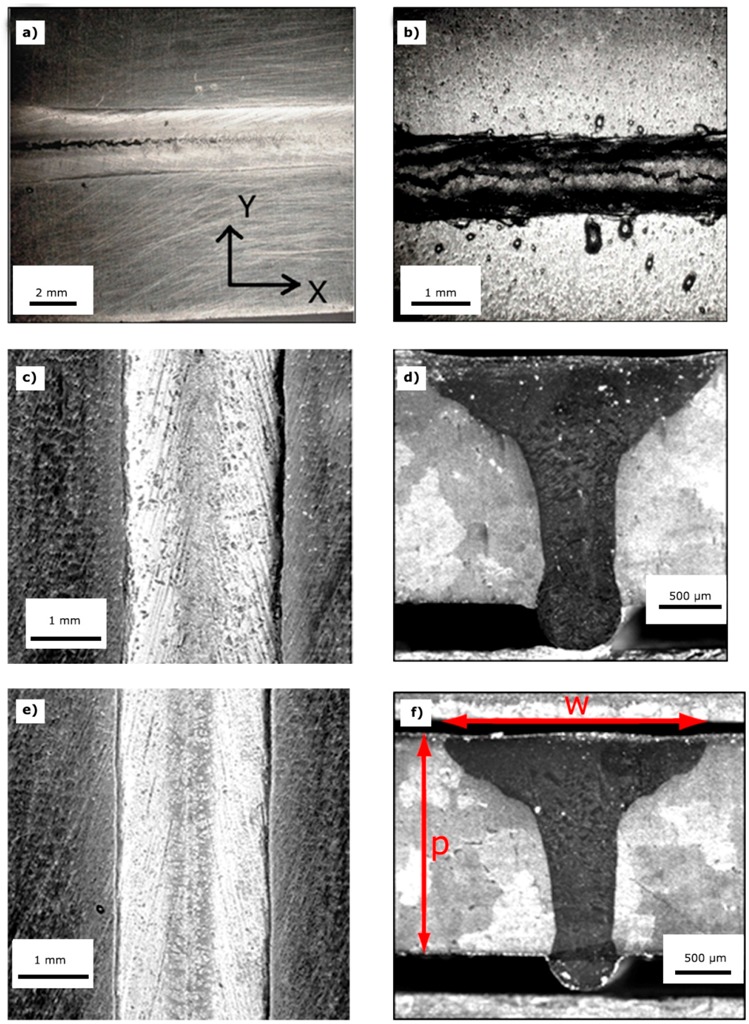
Seams of the samples EB welded with pre-heating (PHT) = 200 °C: pass speed of 1.0 m/min (**a**,**b**); 1.5 m/min (**c**,**d**) and 2.0 m/min (**e**,**f**); the image in (**b**) shows the opposite side of the seam in (**a**); cross-sections of the seams displayed in (**c**,**e**) are shown in (**d**,**f**); respectively; the parameters p and w are displayed in (**f**).

Figure 6a,c,e shows the seams obtained at PHT of 200 °C, and pass speeds of 1.0, 1.5 and 2.0 m/min, respectively. Lower pass speeds involve higher thermal loads, and consequently a larger amount of molten metal which contributes to the formation of hot cracks.

In the case of 1.0 m/min, Figure 6a shows a long crack along the seam axis crossing the plate and emerging on the opposite side (Figure 6b). The cracks are not observed at higher pass speeds (c and e). Furthermore, the comparison between the cross-sections in Figure 6d and f indicates that the aspect ratio p/w (p: depth, w: maximum width) increases with increasing the pass speed (see also Table 2). The aspect ratio describes the shape of the seam; for clarity, the parameters p and w are displayed in Figure 6f.

Considering the results of the tests with PHT of 200 °C, the following tests were performed with PHT of 300 °C by increasing the welding speeds (1.5, 2.0 and 2.5 m/min). Such a change was justified by the better energy adsorption on the work pieces at higher PHT, that allowed full penetration at 2.5 m/min and avoidance of hot cracking. Special attention was paid to liquation microfissures, which often occur in HAZ of Ni-base superalloy welds as the results of incipient melting of MC carbides, γ-γ’ eutectic and complex intermetallics, and are not detectable by X-ray examinations. Therefore, SEM observations at high magnification were conducted to assess the presence of such defects, but no microfissures were revealed.

**Figure 7:**
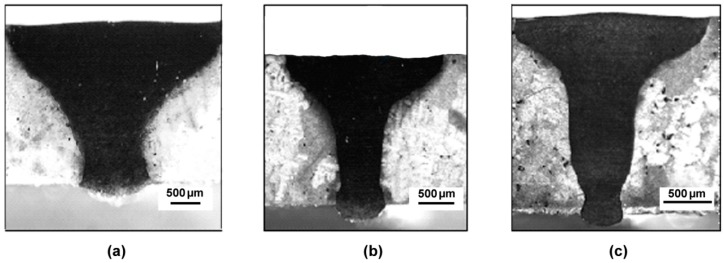
Cross-sections of the seams obtained by EB welding with PHT = 300 °C: pass speed of 1.5 m/min (**a**); 2.0 m/min (**b**) and 2.5 m/min (**c**).

Figure 7a–c displays the cross-sections of seams obtained with PHT of 300 °C, the corresponding values of w and aspect ratios p/w for the examined pass speeds p are reported in Table 2. The aspect ratios were similar to those of cross-sections in Figure 6; however, no cracks were observed in this case for the lower pass speed. Maximum width w and aspect ratio p/w of the joints observed in different conditions of welding pass speed and PHT are reported in Table 2.

**Figure 8:**
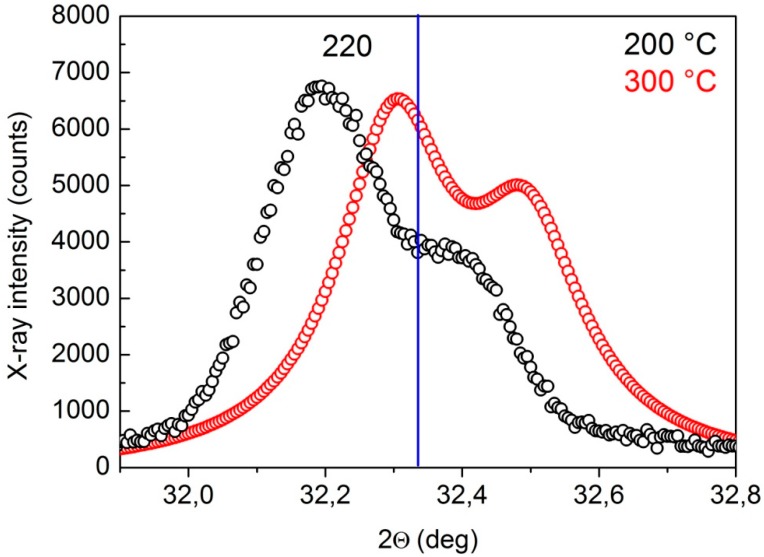
{220} peak profiles of samples welded with PHT of 200 and 300 °C and the same pass speed of 1.5 m/min. The vertical blue line indicates the peak position of base material (BM) free from the stresses arising from welding.

XRD precision peak profiles collected by focusing the beam in MZ show that PHT at 300 °C substantially guaranteed seams that were free of residual stresses for the lowest pass speed examined here. For example, Figure 8 compared the {220} peak profiles of samples welded with PHT of 200 and 300 °C, and the same pass speed of 1.5 m/min. The peak position of the sample welded with PHT = 300 °C almost corresponded to that of BM (no stressed material) as indicated by the vertical blue line, whereas the position of the peak of sample welded with PHT = 200 °C was shifted to lower angles, indicating the presence of internal tensile stresses.

Generally, XRD peak shift arises from a change of lattice spacing that can have different causes, e.g., solid solutioning [33] or defective structures. In the case of IN792 DS, the change arises from stresses of metal solidification which can be so intense that they give rise to cracks. Solidification cracking, which is observed frequently in castings and ingots, can also occur in welding. Such cracking is intergranular or, as in the present case, interdendritic (see Figure 5b). It occurs during the terminal stage of solidification, when the tensile stresses developed across the adjacent grains or dendrites exceed the strength of the almost completely solidified weld metal. The solidifying weld metal tends to contract, owing to thermal contraction and solidification shrinkage. The surrounding metal also tends to contract, but not as much, because it is neither melted nor heated as much on average. Therefore, the contraction of the solidifying metal can be constrained by the surrounding metal thus tensile stresses develop in the solidifying weld metal. On these grounds, the pre-heating of the work-pieces becomes of crucial importance. This phenomenon has been widely investigated, and different models have been proposed. Anyway, all the theoretical models of solidification cracking embody the concept of the formation of a coherent, interlocking solid network that is separated by essentially continuous thin liquid films and thus is ruptured by the tensile stresses.

On the basis of present experimental results, the pre-heating of the plates at 300 °C could be considered a satisfactory solution to realize the EB joints of the IN792 DS superalloy. Therefore, hereinafter only the results obtained from samples realized in this condition will be presented and discussed.

**Figure 9:**
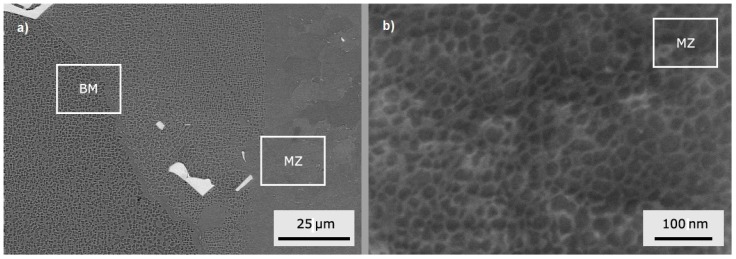
SEM observations of an EB joint (PHT = 300 °C and pass speed *v* = 2.5 m/min): (**a**) MZ microstructure (backscattered electrons imaging); (**b**) round γ’ particles with size ranging from 20 to 40 nm are embedded in the equiaxed grains of MZ (secondary electron imaging).

SEM micrographs in Figure 9a,b show the upward side of the seam (PHT = 300 °C, *v* = 2.5 m/min). The microstructure of MZ was completely different from that of the original material, and consists of nearly equiaxed grains of large size, which formed during the solidification of the melt pool (Figure 9a). In MZ, the γ’ particles had an irregular, rounded shape, and a very small size ranging from 20 to 40 nm (Figure 9b). After solidification the γ’ particles nucleated below solvus (~1120 °C) and, because of the rapid cooling to room temperature, had a very short time to grow, and thus their size was quite small.

The relative amount of γ’ phase in MZ and HAZ of the samples welded with PHT = 300 °C and different pass speeds, was determined through XRD and calculated by Equation (1). The content of γ’ phase in HAZ was substantially the same of BM and is not affected by different welding conditions, thus only the data of MZ are reported in Table 3, together with the average hardness values.

**Figure 10:**
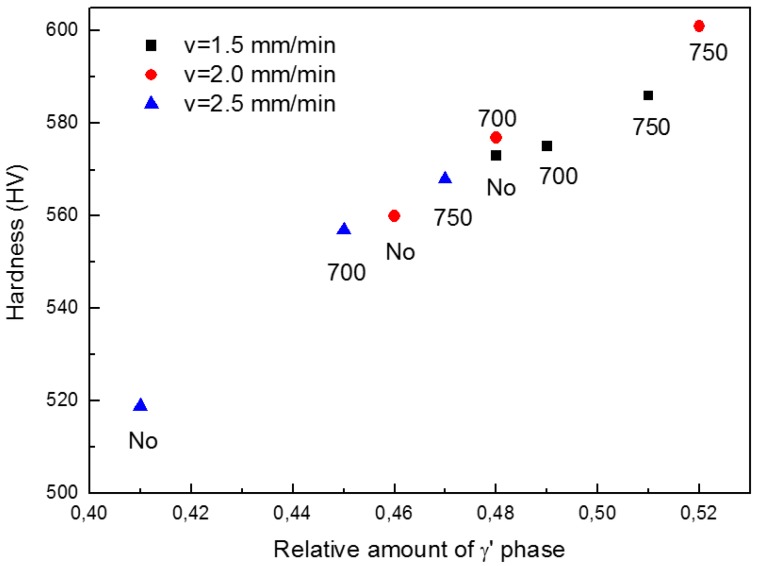
Hardness vs. relative amount of γ’ phase in MZ of samples realized with pre-heating at 300 °C and different pass speeds. The effect of PWHT temperature is also displayed.

The quantity of γ’ phase depends on both PHT and welding pass speed, because these parameters affect the cooling rate of molten and solid metal; in particular the thermal profile below the γ’ solvus curve is of crucial importance because it controls nucleation and growth processes. The faster the cooling rate, the lower the amount of γ’ phase, because of the faster kinetics. The effect of pass speed is strictly connected to the energy transferred from the electron beam to the metal, and is higher for lower pass speeds. Therefore, lower speeds involve slower cooling rates and consequently the formation of larger relative amounts of γ’ phase: 0.48, 0.46 and 0.41 have been measured in the MZ of joints realized with 1.5, 2.0 and 2.5 m/min, respectively. Since hardness substantially depends on the quantity of γ’ phase, similar differences (573, 560 and 519 HV) are observed for the three examined pass speeds. Table 3 also reports the relative amount of γ’ phase and hardness in MZ after PWHTs at 700 and 750 °C of the same samples. For all the different welding conditions, a slight increase of volume fraction of γ’ is found after PWHTs. The increase of the γ’ volume fraction was accompanied by an increase of hardness. To highlight the effects of PWHT temperature and welding speed on the amount of γ’ phase and hardness, data in Table 3 was plotted in Figure 10. The effects of PWHTs will be discussed in the next paragraph.

The high temperature structural stability and properties of Ni base superalloys are strongly affected by chemical gradients due to diffusive processes, which may change the γ’/γ interface energy, driving particle growth and morphology evolution [34,35,36]. Therefore, it is important to evaluate possible segregation phenomena or significant differences of chemical concentration induced by welding process.

**Figure 11:**
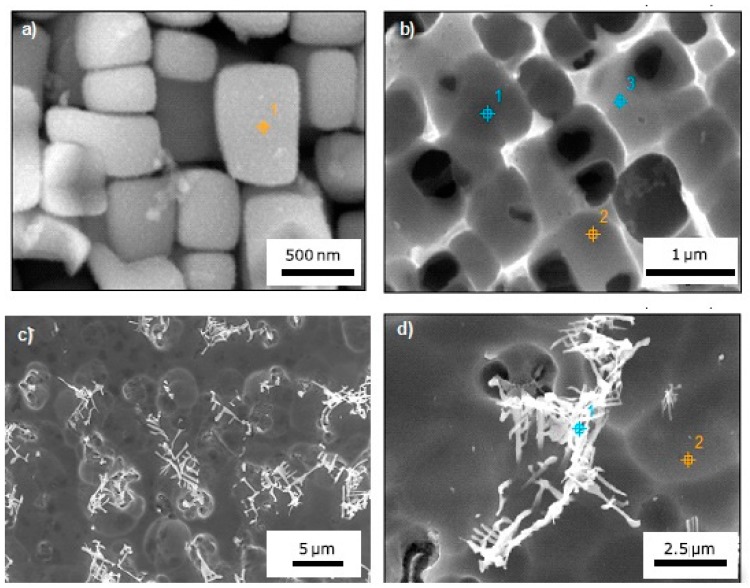
EDS measurements on welds with PHT = 300 °C, pass speed of 1.5 m/min and PWHT = 750 °C: single γ’ particles (**a**); γ phase (**b**) in HAZ; Chinese script structures inside the MZ (**c**,**d**).

EDS measurements were conducted in MZ and HAZ of joints welded under different conditions. Data reported in Table 4 are the average values of at least three acquisitions. A selective electro-etching allowed to perform EDS measurements on welds with PHT = 300 °C, pass speed of 1.5 m/min, and PWHT = 750 °C (Figure 11a,b). The same etching procedure evidenced the presence of complex structures with Chinese script (CS) morphology inside the MZ (Figure 11c,d). CS, which are interconnected strip carbides [37], were also analyzed. It was not possible to detect the chemical composition of γ’ phase in MZ because the particle size was very small and the EDS spatial resolution with an acceleration voltage of 20 kV is not sufficiently high.

As expected, data of EDS analyses in Table 4 show that the γ’ phase in BM was rich in Al, Ti, Ta, and Ni, while higher concentrations of Cr, Co, Mo and W were always observed in the γ phase. The γ phase in HAZ and MZ of joints prepared with different pass speeds exhibited small variations of composition, which were comparable with the experimental errors of EDS measurements. The same considerations can be made for γ’ phase in HAZ. Therefore, EB welding does not induce significant composition changes in γ and γ’ phases.

The γ-carbide eutectic reaction occurred during the final stage of the solidification. The Chinese script structures observed in the MZ were rich in Ti and Ta, with a total content (Ti + Ta) in the range of 11–14 at %, slightly depending on pass speed, while they are poor in Cr and Co content. Such carbide morphology occurs for high cooling rates (>25 μm·s^−1^) [38], whereas for lower cooling rates, carbides appear faceted (see for instance Figure 3d) with a much higher (Ti + Ta) quantity around 85 at %.

The γ-intermetallic eutectic reaction could also occur and carbon content can be used to differentiate metal carbides and intermetallics with high contents of refractory metals such as W and Ta. In future work, the chemical compositions of Chinese scripts will be measured by a electron probe micro-analyzer (EPMA) to evidence the possible presence of complex intermetallics.

The type of carbides and, of course, the specific composition, mainly depends on the cooling rate. The effect of solidification rate on the precipitation and microstructure of directional solidification IN792 DS has been extensively studied by Sun et al. [39], who determined the solidification sequence and the initial precipitation temperature of different phases. They evidenced that script carbides are turned into faceted carbide with a drop in solidification rate, and more specifically Chinese script carbides precipitate when the solidification rate is 50 mm/s, while faceted carbide precipitate when the solidification rate is lower than 5 mm/s.

**Figure 12:**
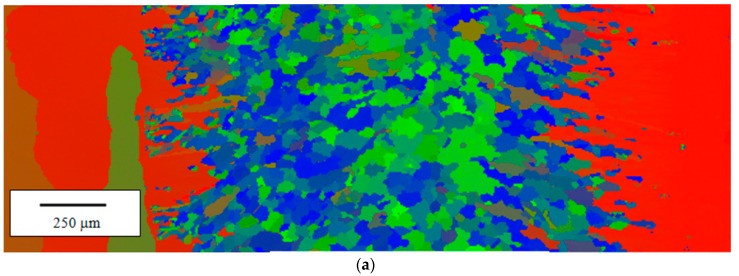

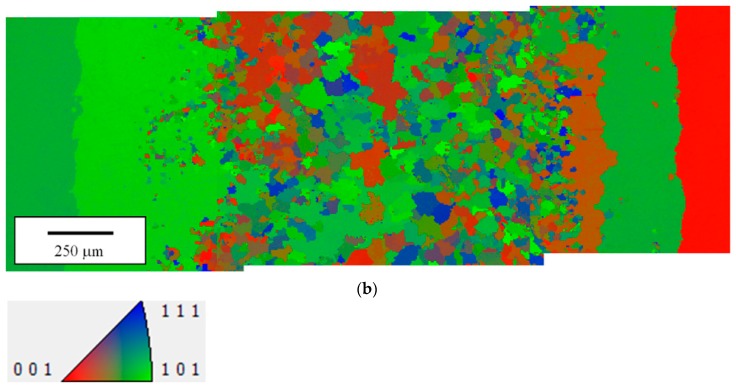
Electron back-scattering diffraction (EBSD) maps collected from joints realized with PHT = 300 °C and pass speeds *v* = 1.5 m/min (**a**) and *v* = 2.0 m/min (**b**).

EBSD analysis of all the samples welded in different conditions confirms that the original columnar structure oriented along the [100] crystallographic orientation is completely lost in MZ. For example, Figure 12a,b shows the EBSD orientation maps of the seams realized with PHT = 300 °C and two pass speeds, *v* = 1.5 and 2.0 m/min: the grains in the MZ exhibit different orientations and the prevalent one is [101] (green), in particular for the welding pass speed of 2 m/min.

The formation of equiaxed grains instead of oriented dendrites depends on the temperature gradient in the liquid ahead the solid-liquid interface which is quite different in EB welding from that of the superalloy production process.

### 3.4. Effect of Post Welding Heat Treatments

**Figure 13:**
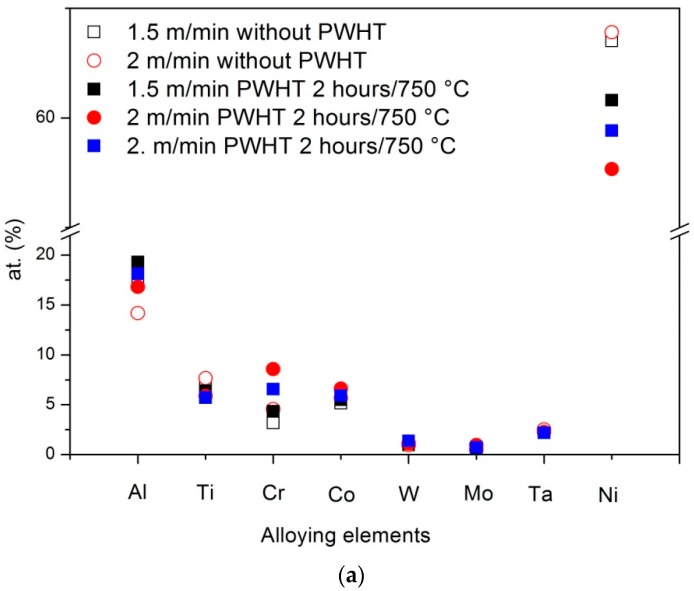

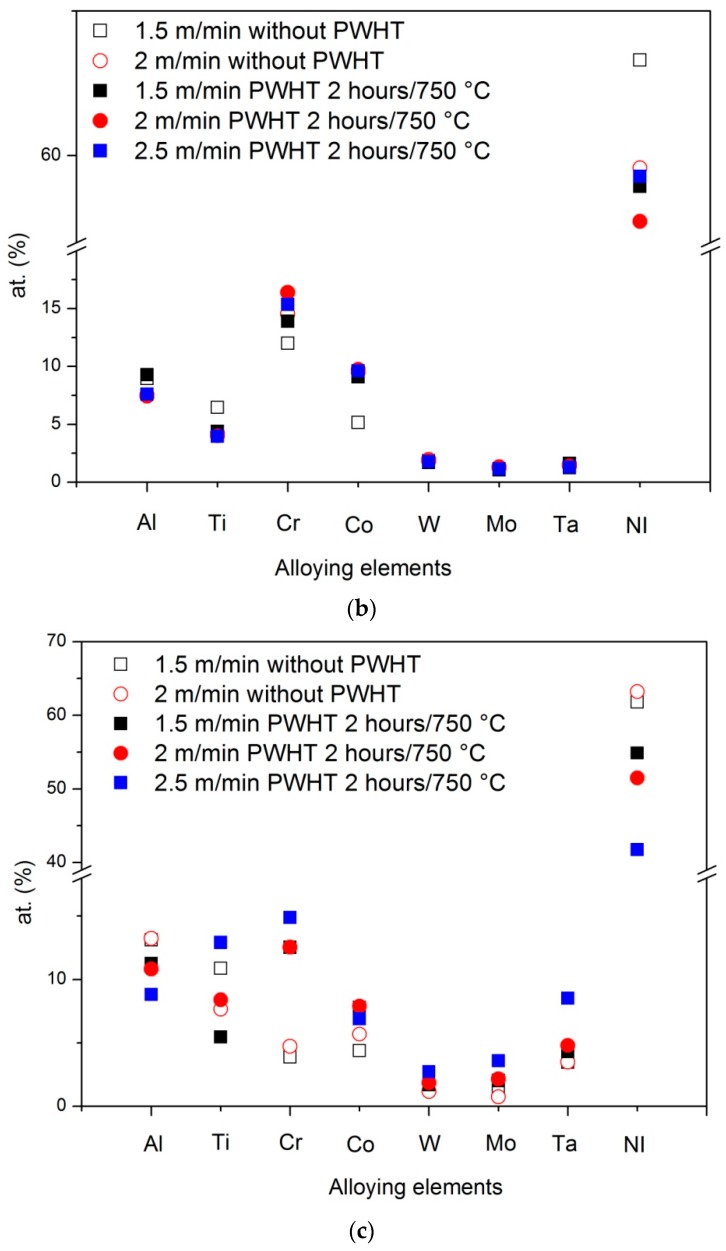
Compositions of γ’ (**a**) and γ (**b**) phases in HAZ and CS (**c**) in MZ (Table 4 and Table 5).

Welded samples were submitted to PWHTs for 2 h at 700 and 750 °C. EDS analyses shown in Table 5 did not indicate significant composition differences of γ’ and γ’ in MZ and HAZ with respect the untreated samples, while the carbides with Chinese script morphology generally exhibit an higher Ta content. In order to highlight the effects of PWHT on samples realized with different pass speeds the compositions of γ and γ’ phases in HAZ and CS in MZ (see Table 4 and Table 5) have been plotted in Figure 13.

After PWHT Ta and W contents of the Chinese script structures increase with increasing welding pass speed. Since carbide size tends to decrease and a more uniform distribution occurs with increasing welding speed the interference of measured carbide compositions by the background is more serious and some composition changes can be attributed to experimental conditions instead of physical processes.

**Figure 14:**
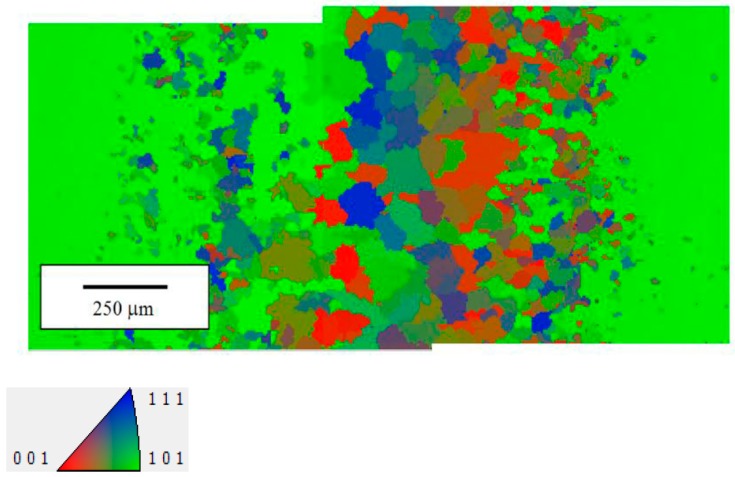
Example for EBSD map collected from a joint realized with PHT = 300 °C and pass speed *v* = 2.0 m/min after PWHTs of 2 h at 700 °C.

An example of EBSD map collected from a joint realized with PHT = 300 °C and pass speed *v* = 2.0 m/min after PWHTs of 2 h at 700 °C is given in Figure 14.

With reference to Table 3, PWHTs at 700 and 750 °C increased the amount of γ’ for all the different welding conditions, and the effect seems to be more pronounced as the temperature increased. From Table 3, there was a strict correlation between the increase of amount of γ’ phase and increase of hardness. In fact, the present results confirmed observations by other investigators [40,41], namely the precipitation of small particles of secondary γ’ from 600 to 875 °C determining an increase of IN792 yield stress and hardness. The phenomenon has been very carefully investigated by Strunz et al. [42] through Small Angle Neutron Scattering (SANS) measurements.

**Figure 15:**
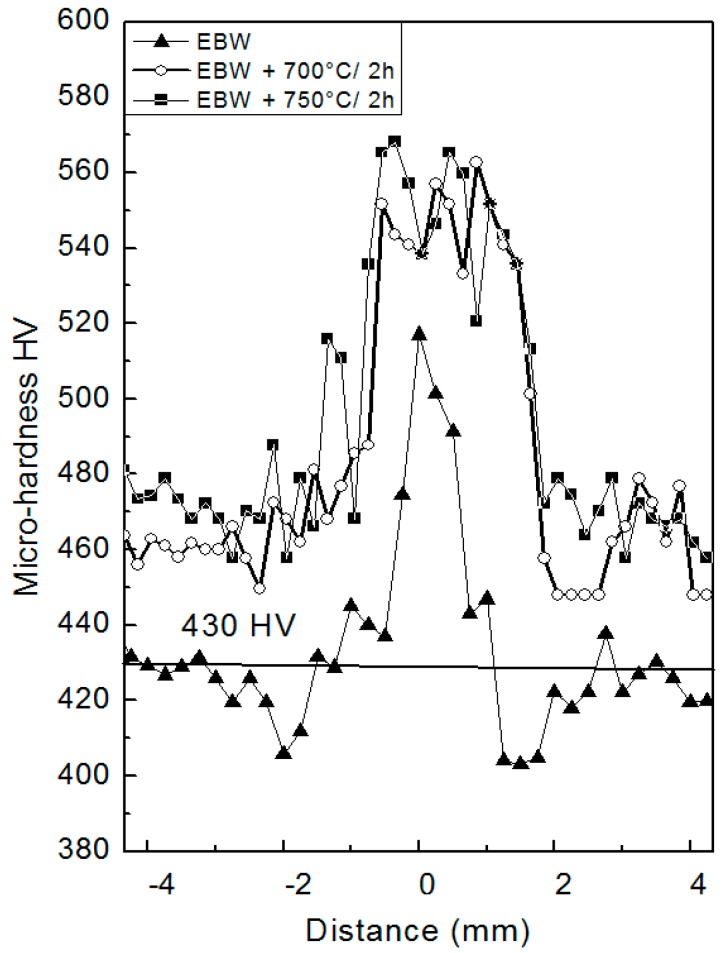
Micro-hardness profiles across a joint realized with PHT = 300 °C and pass speed *v* = 2.5 m/min. Three different conditions are compared: as-welded, after PWHT at 700 °C for 2 h, and after PWHT at 750 °C for 2 h.

Figure 15 compares the micro-hardness profiles across a seam (PHT = 300 °C, *v* = 2.5 m/min), before and after PWHT at 700 °C and 750 °C for 2 h. The horizontal line indicates the mean value of BM (430 HV).

In as-welded conditions, hardness exhibited a mean value of 410 HV in HAZ, and reached a maximum of 520 HV in MZ. The hardness of HAZ was always lower than the horizontal reference line. This trend depended on the specific microstructures in the three weld zones, in particular the γ’ particle size volume fraction and distribution seemed to play a fundamental role in determining the mechanical characteristics. After both heat treatments, the profiles shifted upwards with a remarkable improvement of hardness, while the peak-valley height was a little lower after 2 h at 750 °C, i.e., the mechanical properties across the seam became more homogeneous. As discussed before, the increase of hardness depended on the precipitation of secondary γ’ phase, due to the temperatures of PWHTs. In Strunz et al. [42] it was reported that the precipitation of secondary γ’ in IN792 is maximum at about 725 °C, determining a significant increase of yield stress.

## 4. Conclusions

EB welds were realized on plates of IN 792 DS superalloy by changing the pass speed. The first part of this work evidenced the importance of pre-heating the workpiece to avoid the formation of long cracks in the seam. The results indicate that MZ presented some cracks and porosity if preheating was not applied. The comparison of different pre-heating treatments and pass speeds allowed the identification of the optimal process parameters corresponding to PHT = 300 °C and *v* = 2.5 m/min.

The microstructure of all the joints was then examined by SEM, EDS, EBSD, XRD and Vickers micro-hardness tests. As expected, the structure of directionally oriented grains results were completely lost in MZ. The γ’ phase in MZ consisted of very small (20–40 nm) particles of round shape and its amount depended on both PHT and welding pass speed: lower speeds involved slower cooling rates and consequently the formation of larger relative amounts of γ’ phase. In HAZ, the γ’ phase content was substantially the same as BM. Even if the amount of γ’ phase in MZ was lower than that of the as-received material, the nanometric size of the particles induced an increase of hardness. EDS examinations did not reveal relevant composition changes in γ’ and γ phases.

Finally, the material welded with the optimal process parameters was submitted to two different PWHTs: 2 h at 700 or 750 °C, respectively. Both of them slightly increased the amount of the ordered phase, and the effect was more pronounced at 750 °C, while the size of γ’ particles in MZ remained almost the same; thus hardness profiles measured across the joints shifted upwards, but the peak-valley height was a little lower, indicating more homogeneous characteristics.

## Figures and Tables

**Table:** 

Elements	Al	Ti	Cr	Co	Ni	Mo	Ta	W
**Composition (at %)**	2.27 (±0.36)	46.74 (±0.65)	1.56 (±0.22)	0.83 (±0.09)	5.14 (±0.57)	2.69 (±0.19)	38.66 (±0.52)	2.12 (±0.37)

**Table 1:** Chemical composition of the IN 792 DS superalloy (at %).

C	Al	Ti	Cr	Co	Mo	W	Ta	Ni
0.39	8.88	4.46	14.07	9.13	1.16	1.56	1.46	To balance

**Table 2:** Maximum width w and aspect ratio p/w (p: depth, w: maximum width) of the joints in different conditions of welding pass speed and pre-heating temperature (PHT).

PHT (°C)	Pass Speed (m/min)	Maximum Width w (mm)	Aspect Ratio p/w
200	1.0	3.0	0.50
200	1.5	2.4	0.79
200	2.0	2.4	1.00
300	1.5	3.5	0.54
300	2.0	2.3	0.86
300	2.5	2.0	1.02

**Table 3:** Relative amount of γ’ phase determined by X-ray diffraction (XRD) in MZ of samples welded and hardness with PHT = 300 °C and different pass speeds, before and after post-welding heat treatment (PWHT) at 700 and 750 °C for 2 h.

Welding Pass Speed (m/min)	PHT (°C)	PWHT (°C)	Relative Amount of γ’ Phase in MZ	Hardness in MZ (HV)
1.5	300	No	0.48 ± 0.06	573 ± 25
1.5	300	700	0.49 ± 0.06	575 ± 20
1.5	300	750	0.51 ± 0.06	586 ± 14
2.0	300	No	0.46 ± 0.05	560 ± 20
2.0	300	700	0.48 ± 0.05	577 ± 20
2.0	300	750	0.52 ± 0.06	601 ± 20
2.5	300	No	0.41 ± 0.06	519 ± 15
2.5	300	700	0.45 ± 0.06	557 ± 15
2.5	300	750	0.47 ± 0.06	568 ± 20

**Table 4:** EDS data determined on the phases of BM and of HAZ and MZ in EB joints.

Material Without PWHT-at (%)									
-	Phase	Al	Ti	Cr	Co	Ni	Mo	Ta	W
**BM**	**γ’**	18.00 (±2.23)	6.14 (±0.39)	5.63 (±1.66)	5.82 (±0.62)	60.35 (±1.46)	0.61 (±0.25)	2.25 (±0.19)	1.20 (±0.46)
	**γ**	7.43 (±0.94)	4.53 (±0.46)	15.10 (±1.52)	9.17 (±0.40)	58.89 (±1.34)	1.47 (±0.23)	1.59 (±0.36)	1.81 (±0.09)
**MZ 1.5 m/min**	**Chinese script (CS) structures**	13.12 (±1.14)	10.89 (±0.34)	3.89 (±0.22)	4.39 (±0.13)	61.80 (±1.02)	1.05 (±0.18)	3.45 (±0.22)	1.42 (±0.30)
	**γ + γ’**	6.46 (±0.17)	4.58 (±0.48)	10.89 (±0.26)	8.87 (±1.32)	64.20 (±1.52)	1.47 (±0.25)	1.90 (±0.51)	1.63 (±0.04)
**HAZ 1.5 m/min**	**γ’**	17.06 (±1.12)	7.33 (±0.17)	3.17 (±0.22)	5.15 (±0.19)	63.52 (±0.93)	0.54 (±0.06)	2.26 (±0.19)	0.97 (±0.16)
	**γ**	8.95 (±1.45)	6.45 (±0.14)	11.99 (±0.21)	5.15 (±0.13)	63.30 (±1.48)	1.06 (±0.16)	1.24 (±0.10)	1.85 (±0.36)
**MZ 2 m/min**	**CS structures**	13.23 (±1.05)	7.67 (±0.86)	4.73 (±0.41)	5.69 (±0.27)	63.21 (±1.31)	0.75 (±0.10)	3.50 (±0.34)	1.17 (±0.11)
	**γ + γ’**	7.42 (±0.24)	3.74 (±0.56)	13.33 (±0.26)	9.17 (±0.13)	61.94 (±0.32)	0.89 (±0.06)	1.51 (±0.08)	2.01 (±0.28)
**HAZ 2 m/min**	**γ’**	14.19 (±0.55)	7.66 (±0.23)	4.56 (±0.20)	5.69 (±0.18)	63.91 (±0.78)	0.49 (±0.02)	2.53 (±0.21)	0.98 (±0.13)
	**γ**	7.44 (±0.51)	4.23 (±0.44)	14.53 (±0.33)	9.49 (±0.28)	59.58 (±1.85)	1.24 (±0.74)	1.57 (±0.24)	1.97 (±0.38)

**Table 5:** EDS data determined on the phases of HAZ and MZ in EB joints with PWHT of 2 h at 750 °C.

PWHT 2 h/750 °C-at (%)									
-	Phase	Al	Ti	Cr	Co	Ni	Mo	Ta	W
**MZ 1.5 m/min**	**CS structures**	11.27 (±2.10)	5.46 (±1.40)	12.53 (±1.25)	7.81 (±0.60)	54.88 (±2.64)	2.06 (±1.15)	4.30 (±2.61)	1.69 (±0.63)
	**γ + γ’**	9.18 (±0.80)	4.69 (±0.77)	13.20 (±0.13)	8.89 (±0.49)	59.49 (±1.43)	1.15 (±0.18)	1.84 (±0.30)	1.56 (±0.16)
**HAZ 1.5 m/min**	**γ’**	19.33 (±8.23)	6.41 (±0.95)	4.35 (±0.59)	5.50 (±0.37)	60.81 (±6.79)	0.32 (±0.06)	2.17 (±0.44)	1.12 (±0.15)
	**γ**	9.29 (±4.04)	4.37 (±0.80)	13.90 (±2.17)	9.08 (±0.94)	58.94 (±3.40)	1.12 (±0.23)	1.61 (±0.33)	1.69 (±0.42)
**MZ 2 m/min**	**CS structures**	10.82 (±0.89)	8.41 (±1.26)	12.54 (±0.83)	7.91 (±0.78)	51.48 (2.16)	2.17 (±0.34)	4.81 (±0.91)	1.86 (±0.09)
	**γ + γ’**	9.22 (±2.85)	3.93 (±0.48)	13.22 (±0.31)	9.08 (±0.35)	60.06 (±2.48)	1.00 (±0.17)	1.66 (±0.20)	1.33 (±0.40)
**HAZ 2 m/min**	**γ’**	16.83 (±1.62)	5.91 (±0.37)	8.60 (±2.25)	6.62 (±0.16)	57.67 (±4.68)	0.96 (±0.48)	2.28 (±0.07)	1.13 (±0.09)
	**γ**	7.54 (±0.86)	4.01 (±0.09)	16.38 (±0.71)	9.74 (±0.38)	57.73 (±1.07)	1.34 (±0.20)	1.41 (±0.27)	1.84 (±0.18)
**MZ 2.5 m/min**	**CS structures**	8.81 (±0.96)	12.90 (±0.97)	14.85 (±0.64)	6.89 (±0.66)	41.74 (±2.04)	3.58 (±0.15)	8.52 (±2.88)	2.72 (±0.96)
	**γ**	10.17 (±0.53)	3.97 (±0.97)	13.61 (±0.66)	9.16 (±0.53)	58.87 (±0.64)	1.03 (±0.13)	1.43 (±0.30)	1.76 (±0.30)
**HAZ 2.5 m/min**	**γ’**	18.13 (±3.05)	5.72 (±1.27)	6.56 (±4.87)	5.90 (±1.69)	59.42 (±4.76)	0.71 (±0.38)	2.19 (±0.47)	1.37 (±0.41)
	**γ**	7.60 (±2.06)	3.95 (±0.23)	15.34 (±1.48)	9.61 (±0.28)	59.28 (±3.39)	1.15 (±0.27)	1.30 (±0.08)	1.78 (±0.30)
